# Bioactive Glasses: From Parent 45S5 Composition to Scaffold-Assisted Tissue-Healing Therapies

**DOI:** 10.3390/jfb9010024

**Published:** 2018-03-16

**Authors:** Elisa Fiume, Jacopo Barberi, Enrica Verné, Francesco Baino

**Affiliations:** Institute of Materials Physics and Engineering, Department of Applied Science and Technology, Politecnico di Torino, Corso Duca degli Abruzzi 24, 10129 Torino, Italy; elisa.fiume@polito.it (E.F.); jacopo.barberi@studenti.polito.it (J.B.)

**Keywords:** Bioglass, scaffold, tissue engineering, glass-ceramic, silicate glass, borate glass, phosphate glass, mesoporous bioactive glass, sol–gel, drug release

## Abstract

Nowadays, bioactive glasses (BGs) are mainly used to improve and support the healing process of osseous defects deriving from traumatic events, tumor removal, congenital pathologies, implant revisions, or infections. In the past, several approaches have been proposed in the replacement of extensive bone defects, each one with its own advantages and drawbacks. As a result, the need for synthetic bone grafts is still a remarkable clinical challenge since more than 1 million bone-graft surgical operations are annually performed worldwide. Moreover, recent studies show the effectiveness of BGs in the regeneration of soft tissues, too. Often, surgical criteria do not match the engineering ones and, thus, a compromise is required for getting closer to an ideal outcome in terms of good regeneration, mechanical support, and biocompatibility in contact with living tissues. The aim of the present review is providing a general overview of BGs, with particular reference to their use in clinics over the last decades and the latest synthesis/processing methods. Recent advances in the use of BGs in tissue engineering are outlined, where the use of porous scaffolds is gaining growing importance thanks to the new possibilities given by technological progress extended to both manufacturing processes and functionalization techniques.

## 1. Introduction

The need for replacing damaged parts of the body in order to restore their physiological functionality has always been the driving force which has supported research into the discovery and the design of new materials able to perform this task as efficiently as possible.

After a preliminary definition of biomaterial in the 1950s, mainly based on the criterion of maximum biochemical and biological inertness in contact with body fluids (first-generation materials) [1], the discovery of Bioglass^®^ by Larry L. Hench in 1969 [2] constituted—for the first time in the story of biomaterials—an alternative, extending the concept of biocompatibility to all those materials which were able to promote a positive response of the living system through the formation of a strong tissue–implant bond (second-generation materials) and the genetic activation of specific cell pathways (third-generation smart materials) [3].

Actually, Bioglass^®^ represents the first example of a biomaterial belonging to the third generation, because of the biological role played by its ionic dissolution products, directly released in the physiological environment [4], as well as its capability to strongly bond the host tissue (primarily bone) with the formation of an interfacial calcium phosphate layer [5,6].

Over the last 50 years, numerous studies have been conducted [7,8] to optimize the response of the body to BGs and extend their use to a wider range of specific clinical applications.

Most of this research aimed to define in an exhaustive and satisfactory manner the peculiar features of the material and, in particular, its ability to create a strong bond at the interface with physiological tissues as well as to stimulate tissue healing and regeneration.

BGs offer the possibility of adjusting their composition in a very flexible manner, conferring specific properties to the material, which consequently becomes able to satisfy requirements both in hard and soft-tissue healing applications [9].

The most common medical products based on Bioglass^®^ which are available in clinical practice deal with the regeneration of calcified tissues (e.g., bone, enamel) in orthopedics and dentistry [10].

In addition, technological advances in biomaterials production processes made it possible, in recent years, to fabricate mechanically better-performing 3D structures (scaffolds) [11,12], opening up new possibilities in the replacement of bone tissue also in load-bearing sites [13,14].

Recent studies have also highlighted the enormous potential of BGs in soft tissue repair applications [15,16]. Promising results were obtained with regard to the vascularization process [17] and nerve regeneration [18], as well as in the formation of neo-cartilage [19].

All these aspects make BGs very attractive in the development of TE and regenerative medicine approaches, where the availability of functional artificial in vitro-vascularized tissue substitutes is particularly important in order to guarantee the necessary nutrients and oxygen supply from the earliest days after the device implantation [20].

Given the extension of the topic proposed in the present review, we have analyzed comprehensively the most significant literature available in the field. A Search was conducted in the database SCOPUS by using the following terms as major keywords: bioglass, bioactive glass, scaffold, tissue engineering, sol–gel, mesoporous bioactive glass, composite. The present work provides a wide overview on BGs and BG-based scaffolds for TE, thus representing a valid source of information for researchers interested in a complete and organized presentation from the earlier stages to the latest developments.

## 2. BGs: A Historical Overview

The discovery of BGs in 1969 is attributed to Larry L. Hench, a Graduate Research Professor in the Department of Material Science and Engineering at the University of Florida [21].

**Figure 1:**
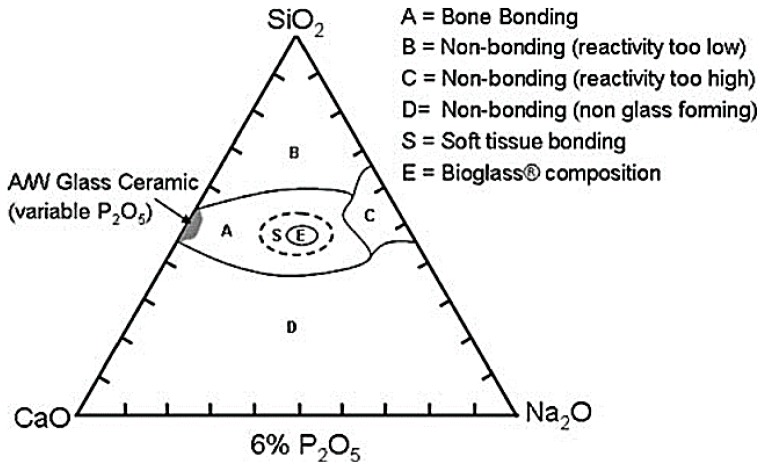
Compositional diagram for bone-bonding. Note regions A, B, C, D. Region S is a region of class A bioactivity where bioactive glasses (BGs) bond to bone and soft tissues and are gene activating. Reproduced with permission from [2].

In order to obtain a material able to survive exposure to the human body with no formation of a scar tissue around the implanted device, from 1969 to 1971, three different glass compositions were tested, on the basis of the Na_2_O–CaO–SiO_2_ ternary state diagram shown in Figure 1 [2].

The optimization process of the glass composition led to the final choice of the so-called 45S5 formulation, 45% SiO_2_–24.5% Na_2_O–24.5% CaO–6% P_2_O_5_ (wt %) [2], characterized by high amounts of Na_2_O and CaO, as well as a relatively high CaO/P_2_O_5_ ratio that make the surface of the material very reactive in a physiological environment [22].

Selected for the high amount of calcium oxide, this composition also had the advantage of being extremely easy to melt due to its proximity to the ternary eutectic [2,23].

The name Bioglass^®^ was trademarked by the University of Florida as the name for the original 45S5 composition and therefore it can be used only with reference to that composition and not generally to indicate BGs [23].

Bioglass^®^ is a silicate glass, thus characterized by the presence of silica (SiO_2_) as a network forming oxide where the basic unit is the SiO_4_ tetrahedron. Each unit shears up to 4 oxygen atoms allowing the creation of the 3D network and connects to each other by –Si–O–Si– bridging oxygen bonds. Sodium and calcium act as network modifiers that disrupt the network creating non-bridging oxygen bonds.

45S5 composition is characterized by enhanced bioactivity and ability to promote stem cell differentiation into an osteoblastic phenotype [24,25], thus favoring the formation of a well mineralized bone matrix [26]. Bioglass^®^ was used over the years to produce scaffolds for the regeneration of bone tissue by several manufacturing approaches (e.g., foam replica method [27], sol–gel foaming [28], bio-templating [29,30], robocasting [31,32,33]), aimed at optimizing both mechanical and bioactive properties of the device.

Thanks to its exceptional properties, the original Bioglass^®^ is still widely used in clinical practice and recent research activities aim to further improve the material performances in order to achieve better and more effective medical treatments.

For example, a new surface modification technique was recently proposed by Meincke et al., who functionalized 45S5 bioactive scaffolds by the heterogeneous nucleation and growth of silver particles directly on the finished product. Surface modification was assessed by FTIR analysis. In vitro results confirmed that the silver coating does not interfere with the apatite-formation process, since a typical cauliflower-like HA layer was observed on the surface of the scaffolds after soaking for 3 days in Simulated Body Fluid (SBF) [34].

Since that moment, many new compositions and other types of BGs have been proposed. In addition to the common silicate glasses, borate glasses are, for example, particularly appreciated for the high dissolution rates, while phosphate glasses are usually characterized by higher solubility once they come in contact with biological fluids.

The first use of Bioglass^®^ in clinics dates back to 1984, when the ossicular chain of the middle ear was restored in a patient who became deaf after an infection that had caused the degradation of two of the three small bones of the middle ear [23].

The second device has been marketed since 1988. It was named ERMI (Endosseous Ridge Maintenance Implant) and consisted of a Bioglass^®^ cone inserted into a fresh tooth extraction site in order to repair the tooth root and provide a stable ridge for teeth [23].

However, the use of such devices was limited by the surgical need for adjustable implants and not already-shaped “rigid” devices. Such monolithic systems are better suited to custom-made applications, expressly designed to meet patient’s needs.

Since the 1990s, Bioglass^®^ was available in the form of particulates or granules, easily adaptable to bone defects as they could be pressed directly inside. Surgeons used to mix particulates and granules with the patient’s blood in order to obtain a putty-like material, easy to inject or spread in order to completely fill the bone defect and allow its expansion within the site [23].

In 1993, PerioGlas^®^ was released by FDA, aimed at the regeneration of the jaw bone resulting from periodontal disease. The particle size ranges from 90 to 710 μm [23]. It was used to heal the bone around the root of a healthy tooth extraction in order to improve bone quality for titanium implant anchoring.

Thanks to the success of particulates in dental practice, in 1999 NovaBone (Nova-Bone Products LLC, Jacksonville, FL, USA) was released, too. It consisted of a bone grafting material in the form of particulate to be used in orthopedic applications in non-loading-bearing sites.

Encouraging results came from the comparison between NovaBone and autografts: the risk of infections and mechanical failure was reduced and the benefit of not having a donor site was remarkable [35].

A significant research program on BGs was launched in Finland in the 1990s that eventually led to the commercialization of particulates of the S53P4 composition, known as BonAlive^®^, which received European approval (CE marking) for orthopedic use as bone graft substitute in 2006 [10].

Nowadays, from a commercial point of view, calcium phosphate-based clinical products are still the leaders in the replacement and repair of bone defects of several extensions [36], because of the difficulty of processing Bioglass^®^ into fibers, scaffolds or surface coatings, as well as long times and high costs associated with clinical trials required to obtain CE marking and FDA approval.

## 3. Interaction of BGs with the Physiological Environment: General Features

### 3.1. Classes of Bioactivity

Considering a generic material intended for surgical implantation, the concepts of bioactivity and biocompatibility are intimately linked as an optimal biocompatibility could be reached by stimulating physiological tissue growth on the surface of the material, which has to be able to support the physiological expected loads.

If a material is able to induce the activation of specific cellular patterns, it has to be considered bioactive.

The formation of an interfacial bond between tissue and implant appears to be the first step towards new tissue formation. The entity and the type of bond are variable according to the type of material used.

The time required for the bond formation is another variable depending on the nature of the material and is related to the bioactivity index, defined as:

(1) IB=100t0.5bb

where $t_{0.5bb}$ is the time required for half of the material surface to establish the bond with the tissue [37].

Given that the final goal is the complete regeneration of the physiological site, there are some important elements which are determinant to establish the host tissue/grafting material bond [38].

Considering bone tissue, the four characteristics that an ideal graft material should exhibit are:

1. *Osteo-integration*, which is the ability to establish a chemical bond with the physiological tissue without the formation of a fibrous layer around the implant [38];
2. *Osteo-conduction*, which is the ability to support the growth of new bone on the surface of the grafting material [39] by allowing the growth of orientated blood vessels and the creation of new Haversian Systems [38];
3. *Osteo-induction*, which stimulates and activates pluripotent stem cells leading to their differentiation to an osteoblastic phenotype [39]. During this process, which is mediated by a signalling cascade, some extracellular and intracellular receptors are involved, the most important of which belong to the TGF-beta superfamily [38];
4. *Osteogenesis*, that involves the synthesis of new bone by osteoblasts that are present within the graft (if cell-seeded biomaterial constructs are used) or have colonized it after implantation [39].

Larry Hench classified materials into two classes of bioactivity on the basis of the level of interaction with the surrounding tissue that they exhibit once implanted [40,41].

- *Class A bioactive materials*: These materials lead to both osteo-conductive and osteo-productive effects because of the rapid reaction mechanism that takes place on the surface of the material and which leads to the dissolution of critical concentration of soluble silica and Ca ions. Class A BGs are able to promote the colonization of their surface by osteogenic stem cells directly inside the surgery-derived bone defect that results in the rapid formation of osteoid bridges between particles, followed by the mineralization of the matrix and the formation of mature bone structures. This phenomenon determines both an extracellular and an intracellular response at the interface because of the interaction with the ions released from the surface [10].
- *Class B bioactive materials*: unlike the previous class, such implants show only osteo-conductive properties determined exclusively by extracellular factors [40]; a typical example is hydroxyapatite.

The 45S5 Bioglass^®^ is an example of class A bioactive material. This glass, in fact, has intracellular effects on the proliferation of bone tissue thanks to the release of soluble silica and other ionic species, but also extracellular effects due to the high surface area and nanometer-scale porosity of the hydrated silica gel and hydroxycarbonate apatite layers which form rapidly [40].

**Figure 2:**
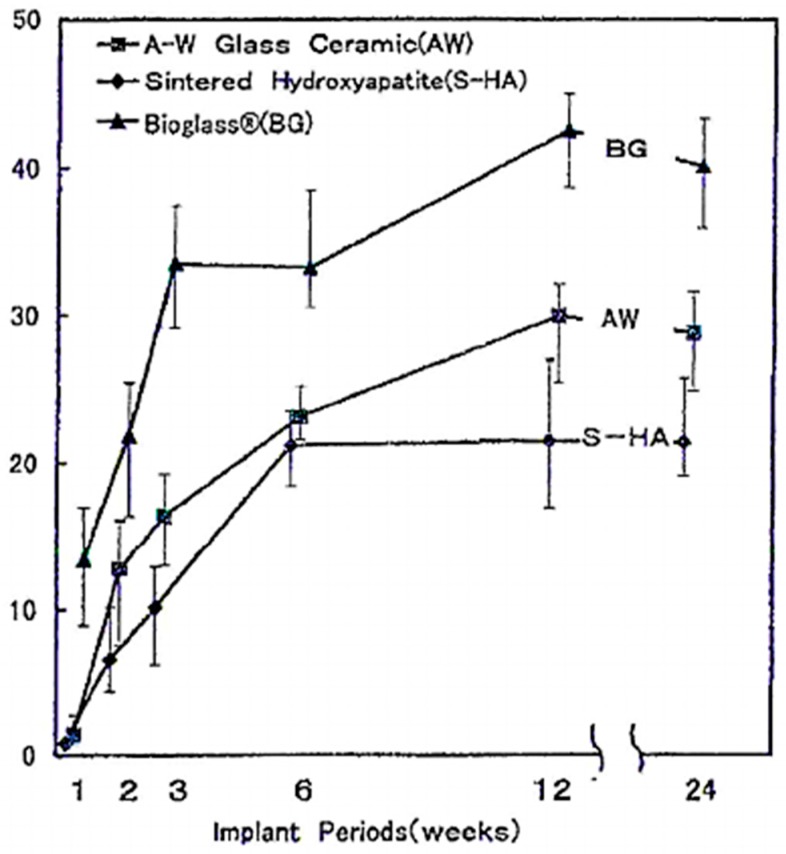
Quantitative comparison of the percentage of bone growth into the bone defect from 1 to 24 weeks owing to 45S5 Bioglass, A–W glass-ceramics and sintered hydroxyapatite (HA) particles of 100–300 µm. Reproduced with permission from [40].

Oonishi et al. showed a correlation between the bioactivity class of the material and the rate of bone formation: class A bioactive material, such as 45S5 Bioglass^®^, promotes tissue formation more than class B materials that dissolve very slowly compared to 45S5 Bioglass^®^ [40]. As Figure 2 shows, the bone growth rate into the inter-particle spaces is higher for BG, with respect to sintered HA and A–W glass-ceramic particles [40].

Some examples of class A and class B materials and the composition–bioactivity class relation are summarized in Table 1 and Table 2, respectively.

### 3.2. Mechanism of Bioactivity: Creation of the Material–Host Tissue Bond

The bioactivity mechanism first proposed by Hench and still accepted for silicate BGs is based on 11 reaction stages which are divided into two different macro-stages [22]:

1. Formation of the hydroxycarbonateapatite (HCA) layer, which takes place during the first 5 steps of reactions, culminating in the crystallization of the amorphous calcium phosphate film [2];
2. Dissolution of ionic products from BG surface and osteogenesis, which leads to the mineralization of the extracellular matrix (ECM) [23].

#### 3.2.1. Formation of the HCA Layer

The first significant event in the formation of the implant–tissue bond turns out to be the formation of a carbonate-substituted hydroxyapatite like (HCA) layer, observed both in vitro and in vivo on the surface of the material, without any significant differences.

In the formation of the said layer, dissolution products coming from the material play a determinant role: in fact, they accumulate in the environment causing local variations of physiological pH and variations in the chemical composition of the solution.

The mechanism of formation of the HCA layer interposed between material and biological fluids involves the first five stages of the mentioned mechanism and they occur ostensibly in sequence [2,23,42,43].

1. Ionic exchange between Ca^2+^ and Na^+^ ions in the material and H^+^ and H_3_O^+^ ions coming from the surrounding environment. Silanol bonds (Si–OH) are established on the surface of the material. An increase of the solution pH is observed due to the release of alkaline ions and a silica-rich layer forms on the surface of the glass. (PO_4_)^3−^ ions are released too, if they are present in the starting composition.
  The chemical reaction is reported below:
  Si–O–Na^+^ + H^+^ + OH^−^ → Si–OH^+^ + Na^+^ (aq) + OH^−^ (2)

This reaction occurs very rapidly, within a few minutes of exposure to body fluids. The surface layer is dealkalisated (alkaline cation-depleted) and it is characterized by a net superficial negative charge.

2. High local value of pH determines the breaking of O–Si–O bonds operated by OH^−^ groups, which cause the breaking of the silica network. Soluble silica is released in the form of Si(OH)_4_ and the silanol groups are exposed on the surface of the material, directly in contact with the solution. The equation describing the mechanism is reported below:
  Si–O–Si + H_2_O → Si–OH + OH–Si (3)

It has been demonstrated that the soluble silica released has a great impact on the proliferation of cells responsible for the formation of new bone in the adjacent area of the glass surface.

3. Silanol groups condensation and re-polymerization of an amorphous silica-rich layer poor in Na^+^ and Ca^2+^ ions. The thickness of this layer varies between 1 and 2 µm.
  It is possible to observe also an increase in the proportion of bridging oxygen during leaching.

The surface is characterized by micro-porosity, with an average pore diameter from 30 to 50 Å and an effective surface area of up to 100 m^2^/g [42].

4. Migration to the surface of Ca ions and phosphate groups through the silica rich layer both from the material and from the solution. On the silica-rich layer a second layer forms, which is composed of amorphous calcium phosphate (ACP). The formation of this second layer was confirmed by surface-sensitive shallow-angle X-ray diffraction (XRD) analysis [23].
5. Hydroxyl and carbonated groups are incorporated from the solution while the process of dissolution of the glass continues starting from the surface. The amorphous layer crystallizes becoming HCA. The resulting surface is very similar to the nano-crystalline mineral phase of the physiological bone tissue both from a compositional and a structural point of view and this allows the direct anchoring of the implant to the living tissue.

The thickness of the HCA layer increases as a function of time up to 100 µm [42]. The compliance of the interface is fundamental in mechanical maintenance of the bond.

As previously mentioned, as the composition influences the formation rate of HCA, a different behavior can be observed within different silica-based systems, with a consistent decrease in bioactivity potential as the silica content increases up to 60 wt % [2,42].

Compositions with low silica content show a less interconnected network that can dissolve rapidly, so that the reactions reported happen in less time. Furthermore, the network connectivity is strongly dependent on the presence of modifiers cations such as Al^3+^, Ti^4+^ or Ta^5+^, which can be optionally incorporated in the glass formulation and can substitute Ca and Na atoms. In this case a reduction in bioactivity is observed as a consequence of the decrease of the dissolution rate [2,23], [42,43].

#### 3.2.2. Ionic Dissolution Products and Osteogenesis

The reaction stages that follow the formation of the HCA layer are not fully understood yet. However, it can be stated that the presence of such a layer promotes protein adsorption, leading to anchorage, proliferation, and subsequent cell differentiation. Once differentiated into the appropriate phenotype, the cells are able to synthesize an extracellular matrix that will be then mineralized [23].

The absorption of organic species such as proteins and growth factors (stage 6) is quite responsible for the characteristics of the HCA layer, since it occurs concurrently with the first four stages [44].

The reaction layer influences the time which macrophages require for the preparation of the implant site to allow the start of tissue regeneration (stage 7) [10]. From the implantation of the material, 12 h are required for the colonization of the surface by stem cells (stage 8) [42]. During stage 9, stem cells proliferate and differentiate into a mature osteoblastic phenotype. Osteoblasts start to produce various growth factors which trigger the cellular attachment as well as mitosis and synthesis of extracellular matrix proteins (stage 10), that lead, after 6–12 days, to the mineralization of the matrix (stage 11) [10]. Table 3 provides a brief overview of the reaction mechanisms described above.

#### 3.2.3. Influence and Genetic Control of the Ionic Dissolution Products on the Osteoblasts Cell Cycle

A strict control on the rates of release of ionic products is fundamental for the material to exhibit a bioactive behavior. If too many ions are released, some toxic effects could be observed. Studies on mature human osteoblasts have shown that the gene expression is highly dose-dependent, and the maximum gene expression is obtained at 20 μg/mL of soluble silica and at 60–90 μg/mL of calcium ions [45]. Similar results have been obtained after the exposure of foetal human osteoblasts to the product resulting from the dissolution of 45S5 Bioglass^®^ [23].

The process of mitosis is essential in the formation of new bone tissue. As a result, it is necessary to provide correct chemical stimuli in order to allow osteo-progenitor cells to enter the active phases of the cell cycle.

**Figure 3:**
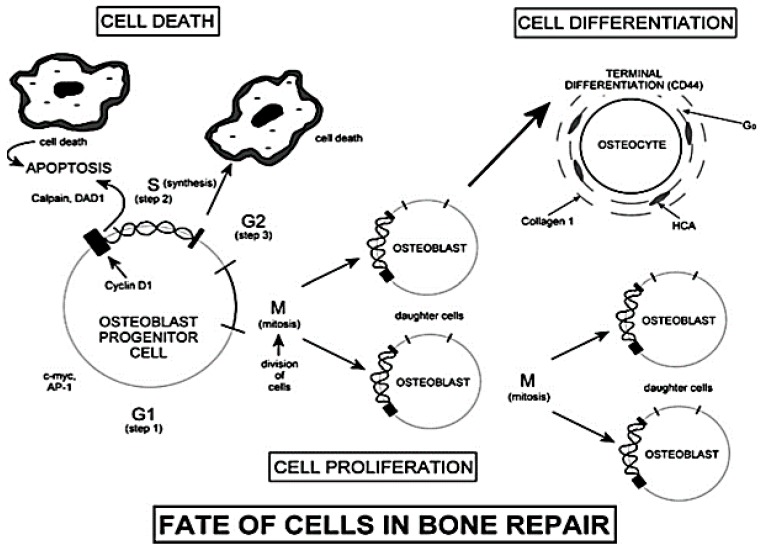
The cell cycle. Reproduced with permission from [2].

It has been found that dissolution products up-regulate the transcription of some factors responsible for the transition from G_0_ phase to G_1_ phase of the cell cycle [23]. According to the scheme shown in Figure 3, the G_0_ phase is representative of a cell resting state. After a period of growth (G_1_ phase), cells enter phase S (synthesis) only if the local chemical conditions show suitable characteristics. With phase S, the synthesis of DNA begins, leading, in some cases, to the duplication of all the chromosomes within the nucleus.

The second phase of growth is called G_2_. If the cell enters the G_2_ phase, the process of mitosis takes place. Before phase G_2_ it is necessary to monitor the synthesis and the activation of several growth factors using dedicated feedback mechanisms [2].

The cellular apoptosis process takes place if the conditions of the surrounding environment are not suitable for the completion of G_1_ and G_2_ phases [2]. Even if the presence of dissolution products increases the number of cells that undergo apoptosis, the surviving cells show an enhanced mitosis and synthesis of proteins and growth factors [23].

The synthesis of collagen follows similar steps during both in vivo and in vitro tests and also the kinetics of bone formation is comparable both in vivo and in vitro [2].

As reported in publications by Xynos and coworkers [26,46], the control on osteoblast cell cycle is achieved by monitoring the release of ions deriving from the dissolution of the glass and in particular the concentration of Si and Ca at the cell–solution interface in order to control the activation of genes responsible for the osteogenesis process [2].

Each ion released has its own effects on the living system:

- Ca^2+^ ions enhance the production of IGF-II as well as the production of glutamate by osteoblasts [4];
- Silicate ions stimulate the production of type I collagen by human osteoblast cells [4].

### 3.3. Effect of Doping Elements and Glass Properties

Furthermore, besides Na^+^ and Ca^2+^, it is possible to include other cations within the glass network in order to confer additional beneficial properties, such as Al^3+^, Mg^2+^, Zn^2+^, Ag^+^ and K^+^ [4,47].

As an example, zinc (Zn) plays an important role in the growth and mineralization of bone both in human beings and animals. In physiological conditions, its level decreases together with aging, skeletal unloading and postmenopausal conditions [48]. Zn positive effect on bone mineralization is associated with its ability to both inhibit osteoclastic activity and enhance protein synthesis in osteoblast cells, while helping to preserve the bone mass [49,50]. ZnO is able to act both as modifier and intermediate oxide according to the content [47]. Higher amounts of ZnO [51,52] lead to a decrease in bioactivity. This aspect has been confirmed by Oudadesse et al. [53], who investigated the apatite formation and cytocompatibility of Zn-containing BGs by comparing pure and 1 wt % zinc-doped BG. Glasses were produced by the traditional melt-quenching route and characterized by bioactivity soaking tests in SBF. Cellular assays were performed in order to assess the toxic potential effect of Zn on L-929 fibroblast cells. It was found that zinc addition has effects both on the bioactivity and the chemical reactivity of the systems since it is able to delay ion release and HCA deposition on the surface of the material. Cytocompatibility was found to be the only function of the size of the particles, directly related to the exposed surface area involved in the ionic exchange [53]. In vitro and in vivo studies indeed confirmed that zinc release turns out to be modest regardless of the content and lower than the human blood plasma value [54]. Zn-doped BGs were also shown to elicit antibacterial effects against various pathogens [4].

Among other beneficial doping elements, the effect of Sr on healthy bone growth, combined with the exceptional potentialities of BGs and glass-ceramics have been already reviewed elsewhere [55]. In the past, the positive effect of strontium incorporation within biomaterials on bone reparation has been observed. Sr-substituted HA coatings have been realized by Capuccini et al. [56], showing enhanced alkaline phosphatase activity and the simultaneous inhibition of the osteoclastic activity, confirmed by Bonnelye et al. who found positive outcomes in the treatment of osteoporotic bone by inducing a steady supply of strontium ions directly to the bone defect site [57]. Later, traditional 45S5 Bioglass^®^ was modified by SrO/CaO substitution in order to evaluate modifications in structure and ionic release [58]. A linear increase in glass density has been observed together with higher dissolution rates in SBF related to an increase of the free volume and the resulting weakening of the network chemical bonds resulting from the strontium substitution [58]. Recently, the same research group used molecular dynamics-based computer simulation to investigate the structure-diffusion-bioactivity relationship in strontium-doped BGs [59]. In this case, a wider range of bioactivity has been analyzed by considering several amounts of silica, from 46 up to 65 mol %. No relevant differences have been found in the network structure of CaO/SrO substituted BGs, suggesting the possibility of opportunely preserving glass properties in terms of physics and chemistry in order to enhance the osteogenic potential of the bioactive system.

According to the promising results obtained as regards bioactivity evaluation in SBF, cellular assays have been performed by exposing Sr-doped sol–gel derived BGs to foetal mouse calvarial bone cells and monitoring ALP activity, OC secretion, and bone markers gene expression by Real-Time Polymerase Chain Reaction (RT-PDLL). The most promising outcomes, related to the high-Sr content (75.5 mol % SiO_2_–1.6 mol % CaO–2.9 mol % SrO) system, confirmed the capability of such material to promote bone regeneration in oral and maxillo-facial clinical applications [60].

In order to confer antimicrobial action to biomaterials, silver was officially approved as an effective agent in the early 20th century [61]. The addition of silver has been reported to also give antimicrobial properties in BG-based systems [62,63,64,65,66]. In particular, the release of silver ions during dissolution acts as killing agent on *Escherichia coli*, *Pseudomonas aeruginosa*, and *Staphylococcus aureus*, with no toxic effect on human osteoblasts and complete bactericidal effect within the first hours of incubation [66,67].

Interestingly, Verné and her coworkers investigated the effect that the Ag-doping technique has on surface properties and interactions with body fluids (SBF) [65]. In particular, they produced Ag-doped melt-derived BGs in the system SiO_2_–CaO–Na_2_O using both ion exchange in molten salts (S-SCN) and aqueous solution (A-SCN). For both S-SCN and A-SCN samples it was found that HA formation was enhanced by Ag^+^ release instead of Na^+^, despite the formation of a thick AgCl layer observed in the first case, which instead affected biocompatibility negatively, having a cytotoxic effect on cells [65].

Recently, Ag-doped BG nanoparticles have been produced by a new modified Stöber method to incorporate higher amounts of Ag^+^ ions in shorter times. Compared to traditional sol–gel processes, antibacterial tests revealed lower effectiveness in terms of inhibition zone, while in vitro bioactivity tests in SBF at 3, 7, and 14 days assessed the lower bioactive potential resulting from the new method [68]. A good antibacterial effect of Ag-doped BG foam scaffolds against Staphylococcus aureus was also recently reported [69].

Outside of skeletal tissue applications, some elements have been found to have a positive effect on angiogenetic processes and wound repair. Among these, copper has been used, for example, to dope a borate BG in order to produce microfibers intended for angiogenesis and healing of critical skin defects. No toxic effect on HUVECs and fibroblasts related to ionic dissolution products was observed in vitro and the gene-expression related to angiogenesis was found to be directly improved by incorporating higher amounts of Cu [70]. Cobalt-doped BGs were also shown to significantly promote angiogenesis in vivo due to hypoxia-mimicking conditions associated to Co^2+^ release [71].

### 3.4. Influence of the Atomic Structure on Dissolution Rate and HCA Nucleation

The design of alternative glass compositions seems to be, nowadays, one of the most attractive challenges in order to confer new properties leading to beneficial outcomes in clinical practice.

The definition of the atomic structure plays a key role in determining the rate of dissolution and the connectivity of the glass network and it could be tailored by considering both the composition and the production technique of the glass.

From a general point of view, high silica content produces a highly interconnected structure where bridging oxygen atoms act as a network stabilizer upon dissolution, determining a lower bioactivity in melt-derived silicate glasses. The addition of network modifier cations was demonstrated to be an effective strategy to improve the bioactivity of melt-derived systems, while phosphate content increases the connectivity and the stability of the network upon exposure to body fluids [23].

It is possible to evaluate the network connectivity of a melt-derived glass directly from the composition by calculating the parameter *N_c_* defined as [72]:

$$N_{c}=\frac{4\left[SiO_{2}\right]-2\left[M_{2}^{I}O+\;M^{II}O\right]+6[P_{2}O_{5}]}{\left[SiO_{2}\right]}$$

*N_c_* (typically described as Q^n^) is related to the average number of bridging oxygen atoms (n) per silicon and can range from 0 to 4 [73], where Q refers to the different structures which can describe network connectivity in a silica-based glass.

It was found that the *N_c_* increases as the bioactivity of the glass decreases. For the original 45S5 composition this value was found to be about 1.9 [73]. If the value exceeds 2.6 the system is not to be considered bioactive [74].

Despite the higher amounts of silica (up to 90 mol %), the high reactivity sol–gel BGs is mainly due to the combination of two factors: textural nano-porosity, which determines a higher specific surface area (SSA), and the action of H^+^ ions, which act as network modifiers by disrupting the silica network [73].

Among the available techniques used to investigate the atomic structure of amorphous systems, magic angle spinning solid-state Nuclear Magnetic Resonance (NMR) allows determining of the relative percentages of Q structures in a glass [23]. Other investigation methods, including computational dynamic modelling, synchrotron-based XRD, and neutron diffraction (ND), are also widely used especially combined with solid-state NMR, Raman spectroscopy, Fourier transform infrared (FTIR) spectroscopy, and computational modelling [73].

## 4. Use and Application Fields of BGs in Clinical Practice

### 4.1. Bone Repair and Orthopedic Surgery

Currently, the gold standard in the surgical treatment of extensive bone defects is represented by autogenous bone grafts that exhibit osteoconductive, osteoinductive, and osteogenic properties [38,75].

Autogenous bone grafts are taken directly from the patient from a donor site [75,76]. The number of osteoblasts that survive the transplantation is not so large but, usually, an adequate number of cells do [39].

Even if the final outcome is good, there are some disadvantages related to the procedure:

1. Two surgical procedures are required, the first for harvesting—which may be painful and stressful for the patient—and the second for implantation;
2. Haematoma formation, blood loss and infections;
3. Arterial and ureteral injuries;
4. Cosmetic defects;
5. Limited availability (bone graft substitute);
6. Chronic pain at the donor site, that has been found to persist for more than 3 months in up to 15% of patients having an iliac graft harvested [38,39,76,77].

An alternative is represented by the use of allografts, which show similar properties to autografts except for the presence of osteogenic cells within the graft, as this graft must be demineralized and deantigenized in order to be accepted by the living system without rejection [39].

Some of the complications associated are fractures, non-union, infections [39], and viral disease transfer. In addition, the immunogenic response as well as the poor capillary infiltration may lead to a delay in the growth of new tissue [38].

Nowadays, other bone grafting synthetic alternatives intended for orthopedic use are commercially available, which differ in composition, mechanism of action, and special features [38].

A further approach which is often used in orthopedic surgery involves the implantation of a metal prosthetic implant previously modified by applying a BG coating on its surface in order to give additional properties—quite different from those of the uncoated device [78]—and guarantee primary stability through the formation of a bonding interface between the bioactive coating and the host tissue [23]. BG coatings are also able to protect the substrate from corrosion, and, in some cases, to avoid the release of potentially toxic metal ions [78].

In such cases it is necessary to carefully consider the degradation rate of BGs since a very high bioactivity could lead to a too-fast dissolution of the coating, resulting in a failure of the treatment [78].

### 4.2. Chondrogenesis and Soft Tissue Repair

Recently, some studies have demonstrated that the dissolution products coming from the degradation of BGs are able to promote not only the osteogenesis process, but also chondrogenesis, which leads to the formation of cartilage [43].

BGs, hydroxyapatite, and hydroxyapatite-glass composite have been compared in order to study the chondrogenetic potential of these materials. In vivo studies on animal models showed that chondrogenesis takes place earlier with BGs than with HA when a bone defect is created in 18 rabbit femurs [19].

Until the 1980s, the research focused on the use of BGs in orthopedics and dentistry for the treatment of bone defects resulting from traumatic events or diseases. The bond between Bioglass^®^ 45S5 and soft connective tissues was first demonstrated by Dr. June Wilson’s research team and the results were reported in 1981 [2]. In particular, it emphasized the strong dependence existing between the nature of the established bond and compositional factors. Referring to the ternary phase diagram shown in Figure 1, it was found that only BGs with greater reactivity (class A) were able to form bonds with soft tissues whereas glasses characterized by a silica content within 52–60 wt % were able to establish bonds only with calcified tissues [2].

In 1982, Bioglass^®^ was used to reconstruct the ossicles chain of the middle ear, showing for the first time the in vivo bond between soft tissues and BGs. In fact, it was found that the material was able to bond directly to both bone and the tympanic membrane via collagen attachment to the surface of the glass [42].

Since then, new scenarios have emerged in the use of BG-oriented research for developing alternative approaches in the field of soft tissue repair, including peripheral nerves, heart, lungs, and ophthalmology. These novel applications have been recently reviewed elsewhere [15,79,80].

## 5. Manufacturing Processes for the Production of BGs

Nowadays, there are two macro-classes of manufacturing processes available for the production of BGs, that is, melt-quenching routes and sol–gel strategies. In this section melt-derived and sol–gel BGs will be described focusing on technological characteristics and general properties.

In order to select the best manufacturing process, several criteria are considered, since the final goal is to obtain a specific composition which would be able to exhibit controlled bioactive behavior, which is the fundamental requirement in clinical applications.

Both the methods listed above lead to the production of bioactive systems, even if some compositional limits have to be considered when talking about melt-derived BGs.

The melt-quenching route, indeed, requires the composition to be opportunely set in order to facilitate the melting and casting into molds shaped according to the final application, and to satisfy the reaction criteria in biological fluids. During this step, an accurate evaluation of the available technologies for the realization of the process is carried out. On the other hand, the sol–gel method allows the expansion of the compositional range without compromising the system bioactivity and overcoming some processing limitations of melt-derived glasses (for example, problems related to the presence of metallic ions forming unwanted alloys with the platinum crucibles do not exist) and permits the use of lower processing temperature (calcination temperatures are lower than melting temperatures).

### 5.1. Melt-Derived BGs

Currently, the US Food and Drug Administration (FDA)-approved melt-derived compositions are 45S5 and S53P4, formed by four different oxides (see Table 1 for detailed information about compositions) [81].

For the production of melt-derived BGs, the synthesis method is similar to that used for common soda-lime glasses. As the final application is clinical usage, a careful standardization of the process is needed and high-purity reagents must be used in order to avoid contamination [81,82].

The melting process of components is carried out at elevated temperatures (typically 1200 °C < T < 1550 °C [81]) using electrical furnaces. The parameters are set in order to guarantee a homogeneous and bubble-free melt. During this stage the temperature is chosen so that the viscosity of the melt can be below 100 Poise [81], which facilitates the bubble elimination. Platinum-covered crucibles are often used for limiting the loss of boron, phosphorous, and fluorine that tend to vaporize during high-temperature thermal treatments.

The time required for the melting (*t_m_*) varies according to the size of the batches and generally, in laboratory experimental practice, 1 h < *t_m_* < 24 h [81]. The melting process may be performed twice or even more times to reach ultra-high levels of homogeneity.

The available forming routes, according to the final product, are as follows:

1. Direct forming via casting into molds, quenching into water, or drawing into continuous fiber;
2. Thermally treating the glass above T_g_ in order to allow the sintering of particles into a porous architecture, drawing of fibers from a pre-form, or sealing particles to obtain coatings on a surface [82].

For the annealing, the thermal treatment is carried out in order to achieve a viscosity value of 10^13^ Poise [81], that allows the elimination of residual stresses originated by the cooling process after forming.

Homogeneous size of powders and granules is obtained by grinding and sifting the glass. During this procedure it is possible to have some equipment-derived contamination.

Crystallization is a critical aspect when considering the compositional range of BGs as thermal treatments, used for example for the sintering process, could induce devitrification. However, it is possible to opportunely design the process parameters by studying accurately the crystallization dynamics of the system in order to obtain bioactive glass-ceramic materials with specific properties. Furthermore, it is possible to control the nucleation of crystals by varying the composition of the glass [81,82].

For example, considering 45S5 and S53P4 BGs, it is very common to have crystallization during the thermal treatment. To limit such a phenomenon, it is possible to add further oxides within the network such as boron oxide, potassium oxide, and magnesium oxide [82].

Also the T_g_ temperature is influenced by composition. It could be determined by both dynamometric and thermal analysis by using experimental relations [81].

The theoretical compressive strength of flawless solid silicate glass is about 35 GPa [82]. If cracks are present, however, the resistance to fracture is very low and even the presence of very small defects could considerably decrease the mechanical strength to values around 14–70 MPa. The Young’s modulus of silica-based glasses is about 45–100 GPa [82].

### 5.2. Sol–gel Derived BGs

Sol–gel glasses are intrinsically nano-porous materials characterized by a high specific surface area (typically above 50 m^2^/g), leading to a bioactivity level and degradation rate significantly higher than traditional melt-derived BGs of analogous composition (specific surface area less than 1 m^2^/g) [83,84].

The sol–gel method is classified as a chemistry-based synthesis route in which the polymerization reaction of a solution containing oxides precursors leads to the gelation of the sol at room temperature [85]. Usually precursors are chosen according to the specific application after an opportune definition of the desired final composition. Maybe the most attractive aspect of the method is the possibility to obtain different products by acting on the processing parameters in a highly versatile way [85].

Sol–gel glasses intended for biomedical application are usually produced by hydrolysis and poly-condensation of alkoxyde precursors (usually tetraethyl orthosilicate (TEOS) and tetramethyl orthosilicate (TMOS)) followed by aging and drying under ambient atmosphere [86,87,88].

**Figure 4:**
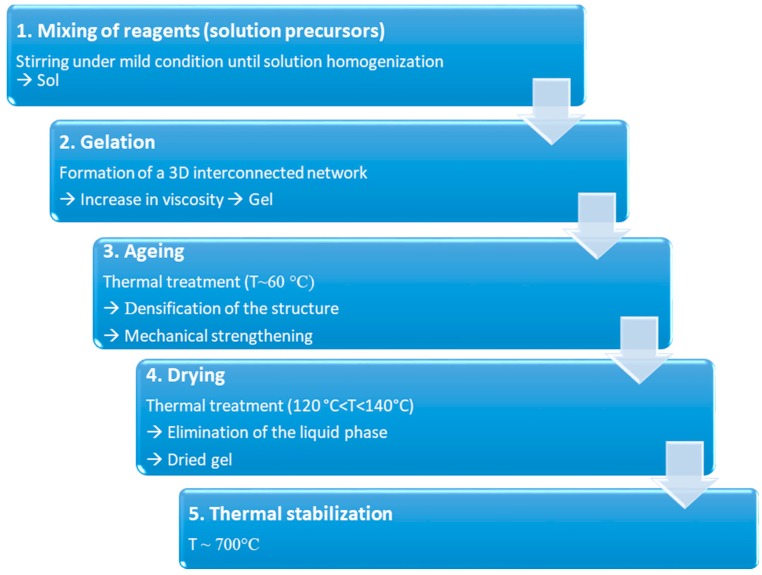
Schematization of the sol–gel process for the production of BGs.

The process could be summarized in 5 different steps, reported in Figure 4.

During step 1, reagents are mixed together at room temperature to induce the formation of covalent bonds between the elements. Hydrolysis and poly-condensation occur simultaneously until homogenization of the solution. Then, during gelation (step 2), viscosity increases due to the formation of a 3D interconnected network. At the end of step 3 (aging), the gel is characterized by a decrease in porosity as well as a remarkable improvement in mechanical strength. Both these aspects are fundamental to avoid cracking during drying (step 4), performed to eliminate the liquid phase from the pores. Dried gel is then stabilized by high temperature thermal treatment, around 700 °C (step 5) [21].

Mesoporous structure is typical of sol–gel materials, as a direct consequence of the synthesis process, with an average pore size of 10–30 nm [89]. Smaller pores with ordered arrangements could be obtained by incorporating supramolecular chemistry in the sol–gel process, which involve the use of a surfactant acting as mesopores template [90], particularly useful in the design of drug delivery systems based on mesoporous bioactive glasses (MBGs) [91].

The mesoporous structure of such materials and their high specific surface area confer on them an excellent apatite-forming ability in simulated body fluids compared to melt-derived glasses, thus expanding the compositional boundaries for BGs [84].

For melt-derived glasses, the maximum silica content allowed to preserve the bioactive potential of the system is limited to 60 wt % [88], while in sol–gel glasses a higher amounts of SiO_2_ (up to 90 wt % [92]) could be included without compromising their bioactivity.

The advantages of using glasses produced by the sol–gel method are remarkable in the biomedical field. Indeed it is possible to functionalize such systems with biomolecules during the formation of the glassy matrix without compromising their physico-chemical properties due to thermal degradation [93]. Furthermore, considering the composition, there is the possibility of simplifying the glass formulation, avoiding the addition of high amounts of Na_2_O to lower the melting temperature and facilitate glass processing [94,95]. Furthermore, doping sol–gel glasses with trace elements is easier compared to melt-quenched glasses and allows preservation of the glass bioactivity while eliciting special therapeutic functions upon ion release (e.g., antibacterial properties, angiogenesis) [96,97].

## 6. BGs in Tissue Engineering

Tissue engineering (TE) and regenerative medicine are modern and smart approaches aimed at repairing and regenerating damaged biological tissues resulting from injuries, pathologies or the aging process. These strategies are based on the development of biocompatible tissue substitutes able to simulate native ECM in order to promote the growth of functional tissue by using both in vivo and in vitro procedures [98].

**Figure 5:**
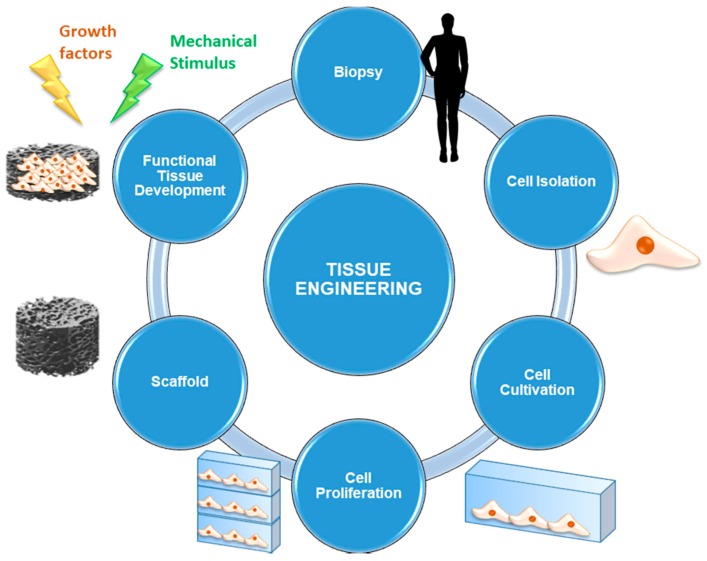
Schematization of a tissue engineering (TE) approach starting from the biopsy and culminating in the implantation of the tissue substitute.

The elements required for implementing a TE and regenerative medicine approach are briefly presented below, while the most commonly-used procedure is schematically shown in Figure 5.

1. Cells are the fundamental element without which the synthesis of new tissue cannot occur. Usually, autologous cells are used: they are directly taken from the site of concern by biopsy in order to avoid the risk of rejection deriving from immune response. Alternatively, it is also possible to use stem cells, that is, undifferentiated cells that are able to evolve to multiple cell lines under the supply of appropriate stimulation protocols. Multipotent stem cells are currently used: they are taken mainly from bone marrow or other tissues, such as the adipose one, easily available in human body.
2. Scaffolds represent 3D (porous) structures that are able to provide physical support to cells by stimulating cell adhesion, migration, proliferation, and differentiation processes. Currently, several types of scaffolds are available, according to the material they are made of. They may be either natural (generally derived from ECM extracted by the patient or by donors, or made by biopolymers) or synthetic (consisting of materials designed ad hoc to mimic the characteristics of the physiological tissue).
3. Signals can be biological, chemical or physical-mechanical. Opportune stimulating procedures are able to influence cell pathways during the processes of proliferation and differentiation by favoring the evolution towards specific phenotypes. These signals are of considerable importance since they are able to ensure cell survival and, therefore, it is necessary that all the cells seeded on the scaffold are affected by them in the same way and with the same efficiency [98].

The main advantage of using a TE approach derives from the possibility of avoiding organ and tissue transplantation, which actually represents a critical procedure due to the problem of rejection and transmission of pathologies, as well as its difficult implementation because of the lack of available organs and tissues that is associated with very long waiting lists for their receival.

At present, TE represents the main field of application of BGs in clinical practice [43], since they perfectly fit into this landscape as materials for the production of functional 3D scaffolds. Despite their poor mechanical properties, newly available techniques for materials processing have made it possible to overcome mechanical limits by developing suitable structures aimed at supporting bone healing process also in load-bearing sites [43].

Before describing the concept of the scaffold in terms of properties, requirements, and production processes, it is considered a useful reminder that understanding tissue biological properties is the first aspect to keep in mind for optimizing the production process of 3D architectures through an appropriate choice of both materials and morphological features.

### 6.1. BG Scaffolds for Bone TE

Musculoskeletal tissues, such as bone and cartilage, have been subject of TE research for a long time and, each year, new experimental protocols and clinical trials are proposed in order to test new materials and new scaffold designs [99]. Since the late 1990s, a great potential has been attributed to the application of BGs in TE and regenerative medicine [100]. Progress over the last 20 years would not have been possible without the introduction of innovative designs and new manufacturing techniques.

**Figure 6:**
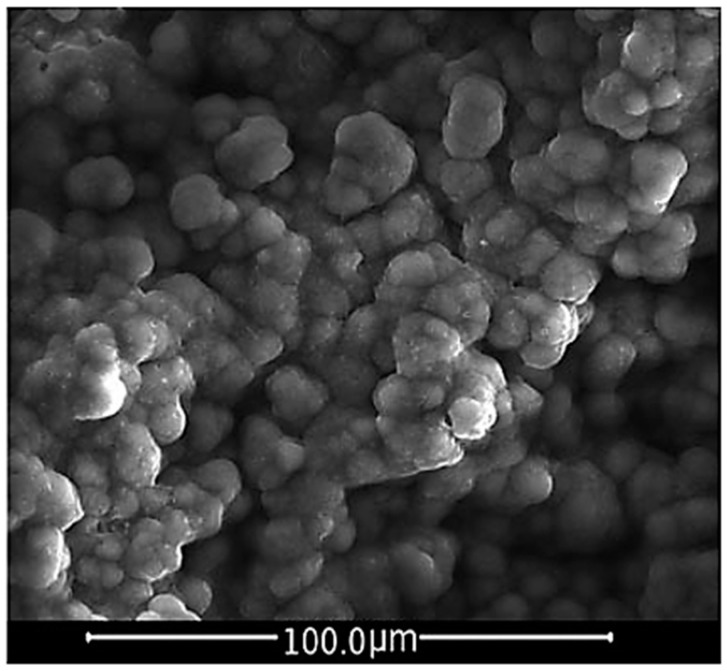
SEM micrograph of a HCA layer precipitated on the surface of a BG sample after 14 days of immersion in SBF. The HCA observed shows a typical cauliflower-like morphology. Reproduced with permission from [101].

Bioceramics, such as hydroxyapatite and calcium phosphates, BGs and related composite materials (made up of bioactive inorganic materials and biodegradable polymers) belong to a class of elected biomaterials for bone TE scaffolds because of their capability to react with the physiological environment in a very effective way by creating a strong bonding interface made up of a bone-like HA layer, resulting in a stable fixation of the material to the host tissue [100]. Figure 6 shows the HCA layer precipitated on a BG sample after a 14-day immersion in SBF.

In general, a scaffold is defined as a 3D device which shows specific physico-chemical and biological properties to induce cell infiltration, adhesion, colonization, and differentiation, until the formation of a functional and mature tissue. In order to properly design a scaffold, it is necessary to be expert in biological processes and tissue characterization, since the scaffold features have to be optimized according to the specific tissue type considered.

As regards hard tissues, for example, an interconnected macro-porosity is required to obtain a rigid structure and promote cell colonization [102].

The requirements of an ideal scaffold for bone TE which would be able to stimulate the growth of tissue (in 3D) are:

1. Biocompatibility and bioactivity The scaffold must not release toxic products within the physiological environment and it must be able to promote anchoring of osteogenic cells that trigger the formation of new bone tissue. After the implantation, the scaffold must produce a negligible immune response in order to prevent the activation of inflammatory patterns which might compromise the healing process [103]. Furthermore, since scaffolds are usually not intended as permanents implants, the constituent materials should exhibit suitable bioactivity and dissolution kinetics comparable to tissue healing rates, in order to allow cells to produce the new extracellular matrix by themselves and permit tissue to regenerate as the scaffold degrades [43];
2. Capability to create a bond with living bone without the formation of a scar layer at the interface [23];
3. Porous and interconnected structure in order to facilitate nutrients exchange, cell migration, and formation of a vascular network to allow tissue oxygenation [104]. An ideal bone scaffold should have an interconnected porous structure, that is, it should be highly permeable with porosity >80–90 vol % and pore diameters in the range of 10–500 μm for cell seeding, tissue ingrowth and vascularization as well as nutrients delivery and waste removal [100]. However, the minimum porosity value admitted is 50 vol %, sufficient to satisfy the necessary requirement for tissue ingrowth [43]. A bimodal pore size distribution has to be preferred in order to mimic the morphologic characteristics of cancellous bone: pores below 50 μm (preferably ≈ 2–10 μm) were found to facilitate the interaction between cells and materials and osteo-integration, while, on the other hand, pores of 100–500 μm enhance new bone formation, bone ingrowth, and capillaries formation (direct osteogenesis) [100];
4. Adaptability in shape and size (mouldability) to completely fill bone defects [23];
5. Suitable degradation rate in order to match the time required for the tissue regeneration and osteoclastic remodeling [23];
6. Maintenance of mechanical properties during degradation and remodeling and load-sharing with host tissue. Ideally, a scaffold should exhibit mechanical properties consistent with the anatomical site of concern. This often represents a clinical challenge considering orthopedics applications. As regards bone, it is necessary to consider the variation of the tissue healing rates depending on the aging process. It is always recommended to consider this aspect together with porosity requirements, since a balance is needed for ensuring stability and integrity of the structure [23,43,100,105];
7. Relatively easy fabrication, production process scalability, and low fabrication costs for large-scale production [23];
8. Sterilization and suitability according to regulations for the usage of biomedical devices [23].

At present, an ideal scaffold for bone TE does not exist (yet). Numerous studies have been conducted in order to optimize the critical aspects that are responsible for the limits in achieving this goal.

Bone is constantly remodeled by load action that occurs during normal daily activities, both in static and dynamic conditions. This suggests that a critical aspect of bone tissue regeneration is the need to have highly mechanically performing materials in order to maintain the physiological function of the tissue.

Considering bone TE scaffolds, there is a very labile balance between the need to employ stiff material able to improve mechanical performance and the need to avoid too high values of rigidity that could lead to tissue resorption caused by the lack of loads transmission between the device and the physiological bone (hypotrophy) [27].

During the regeneration process, the morphological features of the scaffolds, such as microstructure and anisotropy, determine the spatial distribution and the orientation of the newly formed tissue.

Another relevant aspect to be considered is the permeability of the scaffold, which determines how well nutrients permeate the porous structure. Permeability is highly correlated to the interconnection between pores: higher levels of interconnectivity lead to a better perfusion of the whole scaffold allowing cells to receive the same amount of nutrients and oxygen [100].

One additional paramount factor determining the suitability of the scaffold to the regeneration of loaded bone is the influence of the rate of new bone formation on the overall mechanical properties of the implant [82].

In order to provide more information, mechanical behavior of melt-derived BG scaffold will be examined by analyzing the aspects discussed above and how they affect mechanical performance in vivo.

### 6.2. Mechanical Behavior of Silicate BG Scaffolds

From an engineering point of view, it is desirable to select the scaffold material taking into account the mechanical parameters of the tissue of concern. Generally, two ways are considered: (1) selection of the material in order to have a perfect match of mechanical parameters; (2) admittance of some variations of the mechanical parameters within a suitable tolerance range.

Table 4 summarizes the mechanical properties of human cancellous and cortical bone comparing them to dense 45S5 Bioglass^®^ [100].

The limits in mechanical performances of BG scaffolds are related to the intrinsic brittleness of glasses and the interconnected and porous structure required in TE applications. Mechanical failure could occur both during the surgical implantation of the device and during the post-operatory phase, since the dissolution of the material may lead to the formation of cracks responsible for the breaking of the structure even in under-loading conditions [82].

As already reported, crystallization could not always be avoided since a thermal treatment is necessary in scaffold manufacturing, and, as a result, most of the mechanical data available refer to glass-ceramic materials [82]. Densification of the 3D structure upon sintering—which is often concurrent to crystallization—determines the shrinkage of the parent glass “green”, as well as a reduction in porosity and an improvement in mechanical strength [100].

It has been reported that high porosity in glass-ceramic scaffolds results in low mechanical properties. In particular, a highly negative linear relationship between scaffold porosity and compressive strength was found with coefficients of determination R^2^ between 0.80–0.99, obtained by linearly fitting the curve. This means that, for a given scaffold, at least 80% of the variability of compressive strength can be explained by the systematic influence of porosity [100].

**Figure 7:**
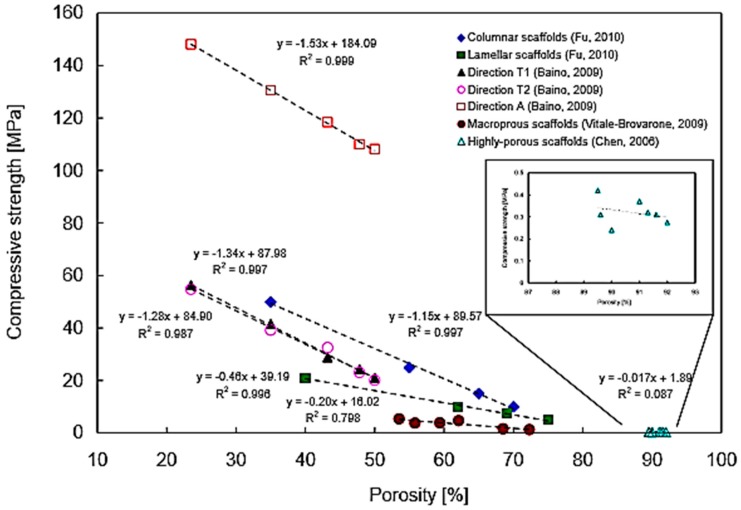
Compressive strength–porosity curve for glass-ceramic scaffolds. The negative slope indicates that an increase in porosity percentage reduces mechanical compressive strength following a linear relationship. However, for very high values of porosity percentage (≈85–95%) the relation cannot be described by a linear curve and the mechanical performances of the scaffold become inconsistent. Reproduced with permission from [100].

Figure 7 shows the relationship between porosity and compression strength in various BG-derived scaffold batches.

**Figure 8:**
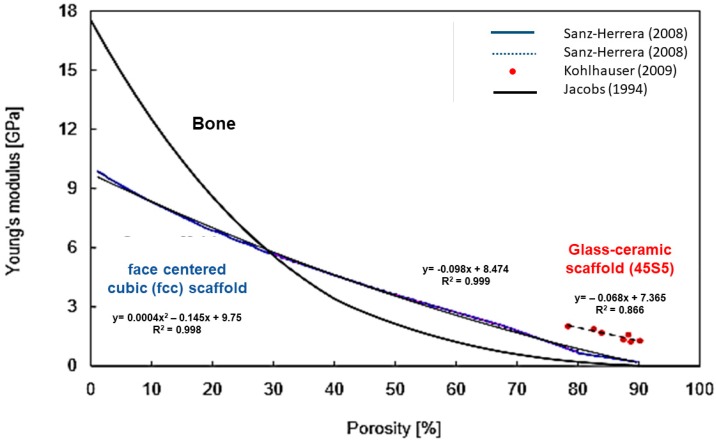
Linear correlation between Young’s modulus and porosity percentage in glass-ceramic scaffolds and comparison with bone. Reproduced with permission from [100].

Figure 8 shows the linear negative correlation between porosity and elastic modulus in BG-derived scaffolds. It can be seen that the relation is not linear in bone tissue, while, for the considered scaffolds, higher linearity is observed in a porosity range between 30 vol % and 80 vol % [100].

Apart from linear correlation, more accurate models exist (i.e., density power law model) and are used to investigate the relationship between porosity percentage and mechanical properties in order to improve the rational design of customized porous bioceramics [106].

Furthermore, it is necessary to observe that the occurrence of crystal nucleation produces a strengthening of the scaffold but, at the same time, it may reduce scaffold reactivity to physiological environment since part of the amorphous structure is replaced by a crystalline phase, which results in a better biochemical stability. Therefore, glass-ceramic scaffolds are usually less bioactive compared to their parent glass counterparts [11].

Recently, the usage of multifunctional BG composite structures has been considered as a possible solution for the mechanical limits discussed above [100].

Conventional composite materials can be produced using a biodegradable polymer matrix with BG particles as a filler phase. The most common polymers used are poly(lactic acid), poly(glycolic acid), and their copolymers, which have been used in clinical applications for many years [23]. In such materials BG acts as strengthening agent by improving the stiffness of the polymer [23]. Sometimes, mechanical properties are improved through the deposition of a polymeric coating onto the surface of the glass and, more specifically, on the scaffold struts in order to make the structure more compliant and tough [82]. Polymer coatings have been applied to highly porous glass-ceramic foam scaffolds with 90 vol % of porosity and pore diameter in the range of 500–700 μm, using poly-DL-lactic acid (PDLLA) or poly(3-hydroxybutyrate) (PHB) [23]. However, a polymeric coating could reduce the bioactive potential of the BG surface because of its covering function. Furthermore, the premature degradation of the polymeric coating associated with the decrease of pH (due to the release of acidic products of degradation) represents another problem in the application of composite materials in vivo [23]. It has been found that mechanical strength rapidly decreases in vivo also because of the interaction between the polymeric coating and the glass, that influence the reciprocal degradation mechanism [82]. It is still very difficult to match the degradation rates of polymers with that of the glass. Mechanical properties are also compromised if there is no strong interfacial bond between the polymeric matrix and BG particles.

A winning strategy was proposed by Liverani et al. [107], who modified 45S5 BG-based scaffolds by dip-coating in a solution with 8 wt % zein in ethanol in order to improve the mechanical strength of the device, thus obtaining increased compressive strength (up to 0.21 ± 0.02 MPa) compared to the uncoated ones. The new coating was compared to collagen I coatings in order to evaluate the outcomes deriving from the use of proteins of different origin. Also, collagen I was found to have a strengthening effect on the structure, even if less effective than the other one. In both cases, bioactivity tests in SBF confirmed that the apatite-forming ability of the material is independent of the presence of the coating [107].

Within the numerous application fields of composite scaffolds, bone regeneration in load-bearing sites represents one of the most claimant clinical challenges.

Various surface modifications of 45S5 scaffolds were investigated by several research groups. In particular, apart from acting on sintering temperature [86,108], microstructure [109,110], and system composition [111], the opportunity to obtain polymer/glass composite scaffolds is a very attractive option [112], typically used to overcome the mechanical brittleness, which, as mentioned above, represents the main limits of such devices.

**Figure 9:**
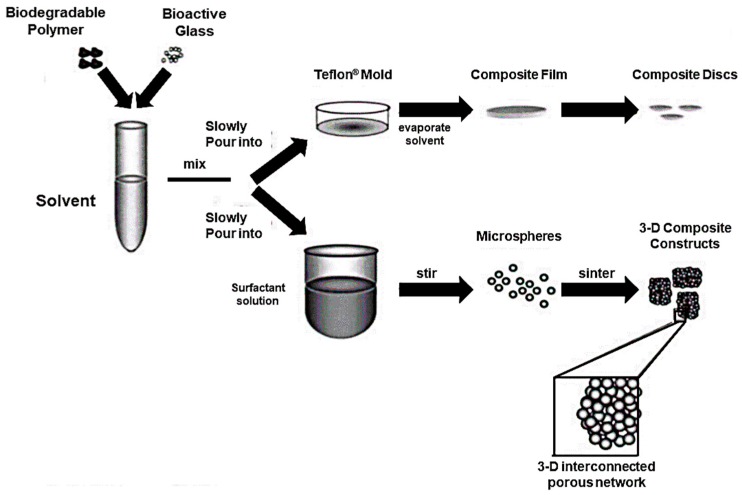
Fabrication process of a composite (PLAGA-BG) of PLAGA and BG. Composites were prepared both in thin film form and as 3D porous scaffolds. Reproduced with permission from [113].

In vitro behavior, cell response, and support of differentiation of the PLAGA-BG composite was deeply investigated by Lu et al. who produced 3D bioactive and biodegradable scaffolds according to the procedure showed in Figure 9.

In this study, mechanical properties of the polymeric phase were successfully increased by the use of BG granules, with elastic modulus increased up to 51.3 ± 6.1 MPa but compressive strength comparable to those of the PLAGA control. Soaking tests in SBF were performed in order to assess the bioactivity of the material and the formation of a calcium phosphate layer was observed within 7 days. Moreover, it was found that the presence of BG granules actually promoted the expression of type I collagen by human osteoblast-like cells (SaOS-2) [113].

Fu et al. recently improved the mechanical performances of 13–93 glass scaffolds under compressive loading conditions by applying a bio-polymeric coating (PCL) on the surface of the device. However, they found that such improvement is limited to compressive strength and does not have the same efficiency for flexural strength and fracture toughness [114].

Even if polymeric coatings are still the most widely used in the reinforcement of BG-3D scaffolds, the use of carbon nanotubes, graphene, and boron nitride nanotubes has also been deeply investigated. Details of such an approach are provided elsewhere [115].

### 6.3. Scaffolds for Bone TE: Design and Manufacturing

Scaffold design and manufacturing are the key steps that translate scaffold-based TE from concept to clinics. Robust experimentation is required for understanding the limits associated with fabrication procedures by using both in vivo and in vitro protocols, aimed at verifying the host tissue response as well as the regenerative power of the device. Several scaffold fabrication techniques, including the foam replication method, salt or sugar leaching, thermally-induced phase separation, microsphere emulsification sintering, electrospinning, computer-assisted rapid prototyping techniques, textile and foam coating methods, have been reported in literature [100].

Progress in material processing recently made it possible to modify surface topography of the scaffold in order to mimic in the best possible way the natural nanostructure of bone, developing what are called biomimetic scaffolds.

Currently there are several methods available, each of which represents a valid means of control of the 3D structure. The most important differences stay in the pore size distribution, pore interconnectivity, mechanical performances, and cost of realization, which is of course an important aspect to mention in order to scale-up the production of the device.

Generally, melt-derived BG scaffolds are produced by (i) sintering the inorganic particles around a template acting as a pore former agent (e.g., polymer sponge or particles), (ii) foaming process, or (iii) solid free form fabrication technologies (SFF).

1. Particles sintering around a template. During the sintering process the glass particles are heated above T_g_, determining their fusion in the contact points. During this procedure the amorphous structure of the glass is preserved by maintaining the temperature below the onset of crystallization. As a result, the definition of sintering window is given as the temperature range limited by T_g_ (lower limit) and T_x_ (upper limit). The sintering procedure turns out to be more effective when the particle size is small enough to avoid the formation of large defects by allowing the creation of a highly sintered and close-packed structure. On the other hand, smaller particles are responsible for higher values of surface area, which result in the enhancement of the crystallization process.

An effective way to control crystal nucleation is to increase the silica content in the glass composition determining the formation of an amorphous and highly interconnected network. However, as already reported, for melt-derived BGs an upper limit exists so that the bioactive potential may be preserved (60 wt % of SiO_2_), and usually the introduction of network modifiers is preferred in order to increase the activation energy of the crystallization process. Some limits of this approach are, as a result, narrow porosity range (40–50 vol %) and low connectivity between adjacent pores [43].

The most common method used for scaffold processing is to sinter particles around a sacrificial template that will be eliminated during the next steps, thus creating the porous structure. Polymer templates; particles; polymeric foams; ice crystals; and corn-, rice-, or potato-derived starch granules can be used as porogen agents [12]. Obviously, each of them influences the morphology of the final porosity. For example, PMMA microbeads have been used as space holders to obtain pores after the sintering process: some limits of this technique are low and uncontrolled pore interconnectivity as well as poor homogeneity in the pore size distribution, since it strongly depends on the PMMA particles size [116]. In order to increase pore interconnection, the sponge replication technique is considered the most effective way to produce suitable bone substitutes. A synthetic or natural sponge-like template is impregnated with a slurry of fine ceramic (or glass) powders and a binding agent, such as colloidal silica or poly(vinyl alcohol) [117]. Excess slurry and powder is then removed from the template pores before sintering by squeezing the sponge. A high-temperature treatment allows the template removal by pyrolysis and the glass (ceramic) particle sintering.

The most crucial steps in the process are the production of a uniform glass coating on the polymeric structure and the risk of pore occlusion by glass particles [118].

Another approach in this family of processing techniques is freeze casting, which uses ice crystals which are removed by sublimation in order to avoid the formation of cracks before sintering. The possibility to control the freezing process allows obtaining scaffolds characterized by an oriented microstructure, resulting in higher mechanical performances in the direction of ice crystals growth. Furthermore, it has been shown that oriented microstructure leads to an enhanced interaction with cells resulting in a better support of proliferation and differentiation processes both in vivo and in vitro [119].

2. Foaming process: Foaming technique aims at the production of 3D highly interconnected porous structures and involves the introduction of gas bubbles into a slurry or sol.

The pore size ranges from 20 μm up to 1–2 mm [12]. The gas could be incorporated within the structure by mechanical action, gas injection or introduction of aerosol propellant. Another approach considers the possibility of developing gas bubbles directly in situ [12].

The porous morphology has to be preserved until high-temperature treatment for the sintering of the struts is performed, so it is necessary to achieve an adequate stabilization of the bubbles by using surfactants that reduce the surface tension at the gas–liquid interface [28]. The action of the surfactants, however, is limited over time.

Melt-derived glasses are foamed by gel-cast foaming, and sol–gel glasses by the sol–gel foaming process, deeply discussed elsewhere [120].

While in the sol–gel foaming the gelation process involves the silica network itself, in the gel-cast foaming, bubbles form upon vigorous agitation of a high-solid-load glass (ceramic) suspension. Specifically, gel-cast foaming is performed by introducing fine melt-derived glass particles into water to produce a slurry that is then vigorously agitated in order to become a foam. A water-soluble monomer can also be added to the slurry; the polymerization process is accelerated by the use of proper catalysts and initiators of the reaction. During the polymerization, a gradual increase in viscosity is observed and, as soon as the gelation point is approached, the foam must be poured into molds. The polymeric phase is then removed by performing a specific heat treatment and final sintering is performed at a higher temperature [121].

3. Solid Free Form Techniques: SFF techniques allow the realization of a design-controlled scaffold manufacturing process. By simply varying processing parameters, it is possible to obtain a precise control on the final 3D structure.

SFF technologies are based on layer-wise manufacturing strategies, since the final 3D structure is obtained by subsequent deposition of material following a bottom-up approach. Precise virtual reconstruction of tissue/organ anatomy can be obtained from clinical images acquired by Computerized Tomography (CT) and Magnetic Resonance Imaging (MRI), allowing the realization of customized devices.

The general process involves:

- Production of a computer-generated model of the wanted structure by the use of a CAD software;
- Segmentation of the model into cross-sections;
- Implementation of the data;
- Production of the physical model [122].

SFF techniques are divided into direct SFF and indirect SFF. Direct SFF involves the direct building of the scaffold from the biomaterial, while, in indirect SFF, the biomaterial is cast into molds that are subsequently removed by using an opportune solvent.

Direct SFF techniques present the great advantage of being highly reproducible thanks to the intrinsic automation of the procedure, even if they are of very difficult implementation because of the compatibility required between equipment and biomaterials; a typical problem, when glass (ceramic) slurries are printed to obtain a layer-wise scaffold, is the risk of nozzle occlusion in the printing head.

Some examples of SFF are 3D-Printing (3DP), Stereo-lithography (SLA), Fused Deposition Modeling (FDM), 3D Plotting, and Phase-change Jet Printing [122].

An electrospinning technique has recently been experimented with in TE approaches to produce nanofibrous scaffolds able to mimic the fibrous structure of the ECM. BG nanofibrous scaffolds exhibit a high surface area, leading to rapid dissolution and conversion to HA, even with high SiO_2_ content in the glass compositions. However, these scaffolds are more suitable for soft TE because of their similarities in morphology with muscles and ligaments, characterized by a fibrous and oriented structure [44,123].

## 7. Towards the Future: The Potential of Borate and Phosphate BGs in TE Approaches

Considering a common TE approach, the necessity of controlling the degradation process of the biomaterial must always be taken into account. Concerning BGs, the rate of degradation has to be opportunely optimized in order to match the times required for the complete healing and regeneration of the tissue itself. In particular, dealing with bone tissue, this aspect is of remarkable importance because of its peculiar remodeling process and constant dynamism [124].

According to this, silicate BGs could show some disadvantages. It is known that, following in vivo implantation of the material, silica-based systems do not undergo a complete conversion to calcium phosphate and times required for the degradation of the material are too long when compared to the physiological growth rate of the tissue [125].

This is the reason why, in recent years, other bioactive systems, based on different forming oxides, have been proposed, among which we count borate and phosphate BGs.

It is common knowledge that boron (B), as an essential micronutrient of living organisms, plays a relevant role in maintaining physiological bone homeostasis, preventing calcium loss and bone demineralization by interacting with several minerals, vitamins, and hormones involved in bone formation [126]. In addition, beneficial effects of boron supplementation on bone mechanical properties have been deeply investigated, as reported elsewhere [127].

According to the available information, some studies have been conducted in order to assess the advantages resulting from the use of both borate and borosilicate glasses in the development of TE approaches as compared to silicate BGs. In particular, it has been shown that B_2_O_3_-based glass systems exhibit a strong bioactivity, reaching complete conversion to a calcium phosphate material after about 200 h immersion in SBF [125,128].

This considerable difference in the degradation rate between silicate and borosilicate/borate BGs can be explained simply considering the structure of the glass network. Because of its coordination number, indeed, boron is not able to fully form 3D structures, leading, from a chemical point of view, to a lower resistance in network interconnections [129].

However, the bioactivity mechanism proposed for borate glasses is comparable to that previously described for silicate-based glasses [42], except for the faster kinetics and the formation of the silica-rich layer, that, in this case, does not take place [43].

The derived advantage is the possibility to design the degradation rate by acting on the composition of the glass which, therefore, can be optimized according to the specific application.

Huang et al. demonstrated the possibility of properly adjusting the conversion rate to HA by replacing SiO_2_ in 45S5 Bioglass^®^ with different amounts of B_2_O_3_, finding that higher quantities of B_2_O_3_ lead to a faster conversion to HA as well as rapid degradation in strength, which limits the application of borate-based BG scaffolds to the repair of non-loaded bone defects [128,130].

**Figure 10:**
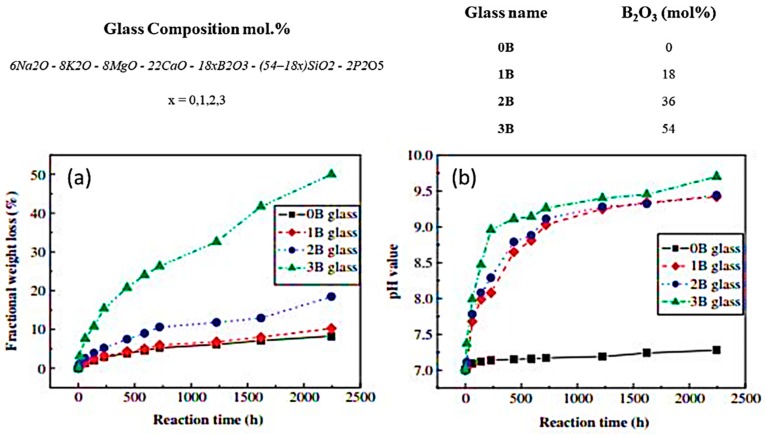
Fractional weight loss (**a**) and pH values (**b**) versus reaction time for particles of the four glasses during immersion in 0.02 M K_2_HPO_4_ solution at 37 °C. Reproduced with permission from [128].

Furthermore, Yao et al. [128] have demonstrated that the reactivity of the glass as well as the rate of conversion to HA is not directly dependent on the B_2_O_3_ content but is rather a function of the SiO_2_ to B_2_O_3_ ratio (Figure 10).

**Figure 11:**
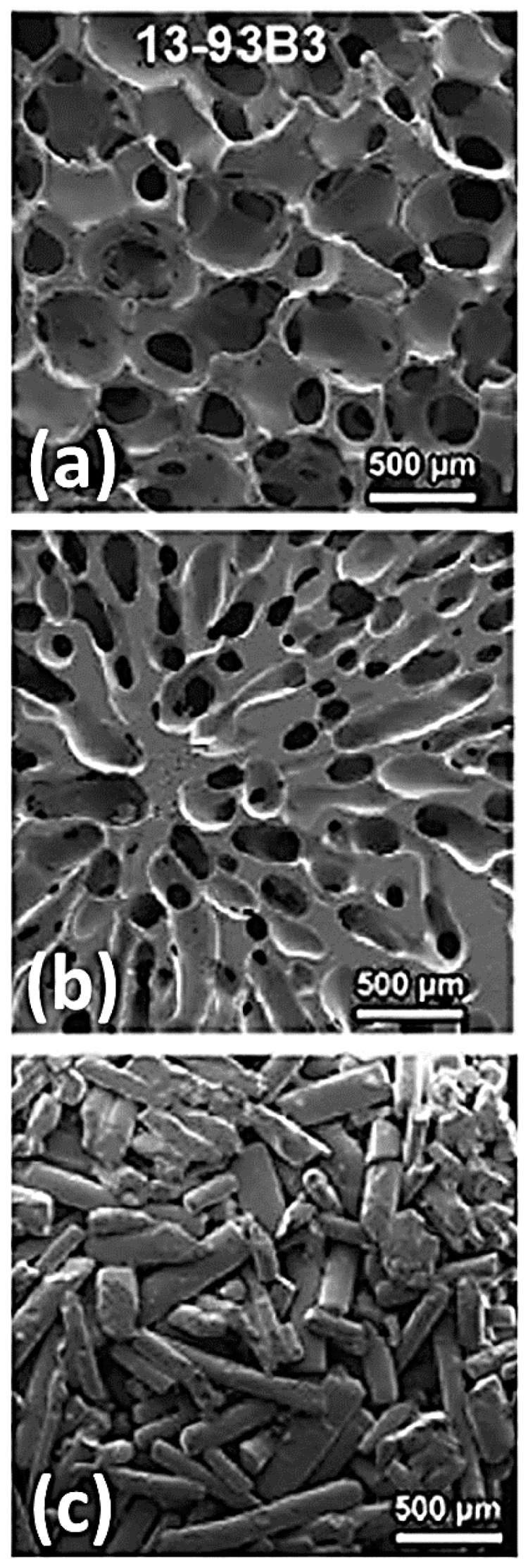
3D borate BG scaffolds with trabecular (**a**), oriented (**b**) and fibrous (**c**) microstructure. Reproduced with permission from [131].

Some in vivo studies demonstrate the importance that scaffold microstructure has in regenerating tissues while preserving the physiological functionality. Borate BGs 3D scaffolds with different microstructures (Figure 11) and porosity between 50 and 70 vol % have been produced by Bi et al. in order to evaluate the formation of new blood vessels, bone ingrowth, and matrix mineralization at 12-week implantation in a rat calvarial defect model [131]. The best outcome, associated with the trabecular microstructure, reveals the importance of following a biomimetic approach in order to enhance bone ingrowth, blood vessel infiltration, and osteconductivity, with 33% new bone formed against 23% and 15% related respectively to the oriented and the fibrous microstructure [131].

Biodegradable borosilicate (13-93B1) BG scaffolds with a trabecular microstructure have been prepared by a polymer foam replication technique and characterized both in vitro and in vivo by Gu and coworkers [132]. Soaking tests in SBF revealed the bioactive potential of the device, with the formation of a poorly crystallized HA thin layer after 30 days. A rabbit femoral head defect model has been used to evaluate the in vivo response of the device at 4 and 8 weeks after implantation, showing a remarkable improvement in bone healing with respect to the unfilled defect, used as a control system [132].

Before implantation, scaffolds have been loaded with platelet-rich plasma and compared to the unloaded ones in order to evaluate the enhancement in bone ingrowth within the segmental defect, thus confirming the effectiveness of using a combined approach to optimize both microstructural features and release of growth factors from the scaffold [132].

Numerous research studies have highlighted the considerable potential of boron-based systems in supporting the proliferation and differentiation of stem cells into the desired phenotype and have been proposed as a carrier for drug delivery systems for the treatment of bone infections.

A winning combination between osteoinductive and drug delivery potential of boron-containing mesoporous BG scaffolds for bone TE has been developed by Wu et al. in order to improve new bone formation by studying the effect of both boron content and dexamethasone (DEX) release on the proliferation, differentiation, and gene expression of osteoblasts in vitro [133].

Borate scaffolds have been produced starting from sol–gel glasses using co-templates of nonionic block polymer P123, to produce meso-porosity, and polyurethane sponges, to create macro-pores. Regardless of boron content (varying from 0 up to 10 mol %), a DEX sustained release (up to 350 h) has been observed in all the cases, with no relevant differences in the release kinetics. The study shows that the loading efficiency of the scaffold is directly related to the porosity as a direct function of the specific surface area (194–265 m^2^/g). Controlled boron ion release has been found to have a positive effect on cell proliferation and ColI and Rux2 expression, while the release of DEX is directly responsible for enhanced ALP activity and gene expression related to osteogenesis processes [133].

Borate BG implants loaded with teicoplanin have been successfully developed by Zhang et al. for the treatment of chronic bone infections in a rabbit model. The conversion of the device to a porous HA-type graft has been previously assessed by in vitro soaking tests in SBF. In vivo evaluation of the device confirmed the beneficial effect of the sustained release combined with the enhanced bioactivity of borate systems, with no toxic effect related to the ion release within the physiological environment [134].

Recently, on the basis of the promising results obtained both in bone repair and drug delivery [132,133,134,135,136,137], borate-based melt-derived glass (BG) fibers have been proposed also for soft TE applications as wound dressing material aimed at supporting dermal repair.

Chen et al. successfully demonstrated the capability of borate-based glass fibers to enhance vascular endothelial growth factors (VEGF) secretion, responsible for better epithelization and collagen deposition, under dynamic flow conditions [138]. Another study, conducted by Zhou et al., compares borate BG micro-fibers and 45S5 Bioglass^®^ (SiG) micro-fibers, confirming the better biodegradation and bioactivity behavior of borate systems, with no ion released-derived toxic effect on HUVECs in vitro. Consistently, in vivo tests show the capability of such systems to promote angiogenesis and dermal regeneration, with about 65% of wound closure reached after only 7 days of treatment, against 61% related to SiG micro-fibers [139].

A commercial product was also recently developed by Mo-Sci Corporation (Rolla, MO, USA) for wound healing [140]. Biodegradable borate glass nanofibres (“cotton-candy”) mimicking the microstructure of a fibrin clot were reported to accelerate wound healing in both animals and human patients [141]. These BG fibres (DermaFuse™/Mirragen™) have a high content of calcium, which accelerates the migration of epidermal cells and therefore promotes skin regeneration.

One aspect not to be underestimated, however, remains the toxic potential of boron in contact with physiological tissues. This issue was pointed out in some in vitro studies but the results could not be representative of what actually occurs in an in vivo situation with fluid circulation. On the other hand, it was shown that toxicity of boron may be significantly reduced under dynamic culture conditions, which closely approach an in vivo-like situation [43].

In phosphate based systems, P_2_O_5_ acts as network former oxide, while CaO and Na_2_O are network modifiers. Phosphate glasses are categorized as class A bioactive material with tunable properties according to composition modifications [44].

**Figure 12:**
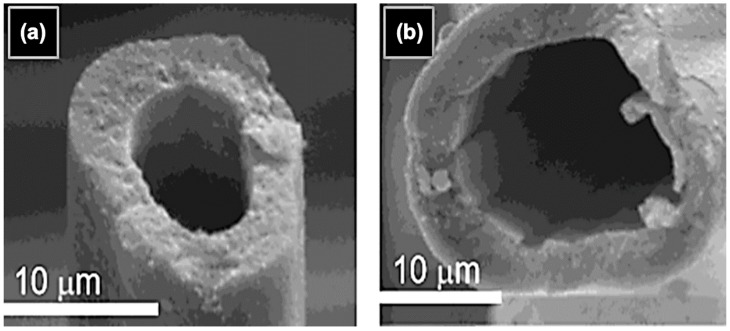
SEM images showing the tubular structures formed from glass fibers after 18 months of degradation; (**a**) 3 and (**b**) 5 mol % Fe_2_O_3_ containing glass fibers respectively. Reproduced with permission from [44].

Just like silicon, the building unit of phosphate glasses is tetrahedral too, but phosphorus can only share three out of its four oxygen atoms because it nominally has charge of 5^+^, while silicon has a charge of 4^+^ [44]. The oxygen atoms that are not shared form a terminal double bond with the central phosphorus atom. As a result, phosphate BGs are characterized by a very high level of flexibility in the orientation of tetrahedron when compared to silicate BGs. The advantage of processing them into fibers (Figure 12) makes them very suitable for soft TE applications for the regeneration and the repair of muscles, tendons, and ligaments, which show a high anisotropy in their organization [142]. These fibers could also be incorporated into bone cements for the fixation of orthopedic devices [44]. Other application fields involve drug delivery and cell transfer, in which fibers could support the nerve healing process by allowing cell migration [44].

It is also possible to incorporate, within the same device, fibers characterized by different degradation rates in order to perform more than one task at the same time (i.e., assistance in the vascularization process and alignment of cells) [44].

In addition, such systems show a very high affinity with bone because of phosphates, which are naturally present in the organic mineral phase of the tissue. This material shows its enormous potential in the realization of absorbable grafts due to its rapid solubility, although its bioactivity is not so pronounced [43].

## Figures and Tables

**Table 1:** Bioactivity properties and compositions of some BGs and glass-ceramics intended for medical use and dental applications. Data from [10].

Composition (wt %)	45S5 Bioglass (NovaBone)	S53P4 (AbminDent 1)	A–W Glass-Ceramic (Cerabone)
Na_2_O	24.5	23	0
CaO	24.5	20	44.7
CaF_2_	0	0	0.5
MgO	0	0	4.6
P_2_O_5_	6	4	16.2
SiO_2_	45	53	34
Phases	Glass	Glass	Apatite Beta-Wollastonite Glass
Class of Bioactivity	A	B	B

**Table 2:** BG composition ranges (wt %). Data reported are related to melt-derived BGs. Data from [42].

	Class A Bioactivity (wt %)	Class B Bioactivity (wt %)
SiO_2_	42–50	52–58
Na_2_O	14–28	3–20
CaO	12–26	8–20
P_2_O_5_	3–9	3–12
Al_2_O_3_	0–1	0–3
MgO	0–3	0–12
K_2_O	0–6	0–12
CaF_2_	0–12	0–18

**Table 3:** 45S5 Bioglass^®^ reaction stages related to time increase.

Stage	Reaction Event
**11**	Crystallization of matrix
**10**	Cellular attachment
**9**	Differentiation of steam cells
**8**	Attachment of steam cells
**7**	Action of macrophages
**6**	Adsorption of biological moieties
**5**	Nucleation and crystallization of calcium phosphate to HCA
**4**	Precipitation of amorphous calcium phosphate
**3–2**	Dissolution and re-polymerization of surface silica
**1**	Ion exchange
**0**	Initial glass surface

**Table 4:** Mechanical properties of trabecular and cortical bone compared to 45S5 BG composition. Data from [100].

Material Property	Trabecular Bone	Cortical Bone	45S5 Bioglass
Compressive strength (MPa)	0.1–16	130–200	500
Tensile strength (MPa)	n.a.	50–151	42
Compressive elastic modulus (GPa)	0.12–1.1	11.5–17	n.a.
Young’s modulus (GPa)	0.05–0.5	7–30	35
Fracture toughness (MPa·m^1/2^)	n.a.	2–12	0.7–1.1
